# Foreign Body Response to Neuroimplantation: Machine Learning-Assisted Quantitative Analysis of Astrogliosis

**DOI:** 10.3390/ijms27083524

**Published:** 2026-04-15

**Authors:** Anastasiia A. Melnikova, Anton A. Egorchev, Alexander A. Rosin, Leniz F. Nurullin, Nikita S. Lipachev, Daria S. Vedischeva, Dmitry V. Derzhavin, Stepan S. Perepechenov, Ekaterina A. Sukhodolova, Gleb V. Shabernev, Angelina A. Titova, Ramziya G. Kiyamova, Andrey P. Kiyasov, Dmitry E. Chickrin, Albert V. Aganov, Dmitry V. Samigullin, Irina Yu. Popova, Mikhail Paveliev

**Affiliations:** 1 Institute of Physics, Kazan Federal University, Kremlyovskaya 16a, Kazan 420008, Russia; nikita.lipachev@gmail.com (N.S.L.); dvedom24@gmail.com (D.S.V.); d.sukhodolova@gmail.com (E.A.S.); alvaraganov@gmail.com (A.V.A.); 2 Institute of Computational Mathematics and Information Technologies, Kazan Federal University, Kremlyovskaya 35, Kazan 420008, Russia; anton@egorchev.ru (A.A.E.); alearosin@kpfu.ru (A.A.R.); dmitry.derzhavin@gmail.com (D.V.D.); gleb@it.kpfu.ru (G.V.S.); 3 Kazan Institute of Biochemistry and Biophysics, FRC Kazan Scientific Center, Russian Academy of Sciences, Lobachevskogo 2/31, Kazan 420111, Russia; leniz2001@mail.ru (L.F.N.); samid75@mail.ru (D.V.S.); 4 Department of Medical Biology and Genetics, Kazan State Medical University, Butlerova 49, Kazan 420012, Russia; 5 Institute of Fundamental Medicine and Biology, Kazan Federal University, Karl Marx 74, Kazan 420015, Russia; ssperepechenov@gmail.com (S.S.P.); anjerika@list.ru (A.A.T.); kiyamova@mail.ru (R.G.K.); kiassov@mail.ru (A.P.K.); 6 Institute of Theoretical and Experimental Biophysics, Russian Academy of Science, Institutskaya 3, Puschino 422290, Russia; irushaster@gmail.com; 7 Institute of Artificial Intelligence, Robotics and Systems Engineering, Kazan Federal University, Kremlyovskaya 18, Kazan 420008, Russia; dmitry.kfu@ya.ru; 8 Department of Sports Engineering, Kazan National Research Technical University Named After A.N. Tupolev-KAI, 10 K. Marx St., Kazan 420111, Russia

**Keywords:** brain–computer interface, neuroinflammation, artificial intelligence, glia, random forest, gliosis

## Abstract

Neuroimplants represent an emerging medical technology, offering new therapeutic approaches for severe neurological and psychiatric disorders. One of the key limitations to long-term neuroimplant performance is the foreign body response elicited by intracortical implantation. Among the contributing cell types, astrocytes play a central role in glial scar formation around the implant, which can compromise device functionality. Immunofluorescence of glial fibrillary acidic protein (GFAP) provides a well-established marker of astrogliosis (neuroinflammation), yet quantitative and reproducible assessment of astrocyte morphology remains challenging due to the complexity and variability of image analysis approaches. Here, we aimed to quantitatively assess implantation-induced astrogliosis and to determine how classifier training strategy influences segmentation outcomes and morphometric measurements. We present a machine learning-assisted pipeline based on the LabKit plugin in Fiji for segmentation and morphometric analysis of GFAP-positive astrocytes in peri-implant scar versus distant cortical regions. Using this approach, we demonstrate an increase in GFAP expression, cell area, and astrocytic process length as well as the redistribution of GFAP signal along astrocytic processes within scar regions. We show that different classifier training strategies produce systematically distinct segmentation outcomes, with rule-compliant annotation improving agreement with manually defined ground truth. These findings highlight the critical role of annotation strategy in shallow learning-based segmentation and provide a practical framework for improving reproducibility of astrocyte morphometry in studies of neuroinflammation and neuroimplant biocompatibility.

## 1. Introduction

Brain-computer interfaces represent a rapidly developing branch of medical technology with the potential to transform the treatment of severe neurological and psychiatric disorders [1,2,3]. Invasive interfaces, in particular, provide the spatial and temporal resolution required for neuroprosthetic applications and high-fidelity neural recording [4,5]. However, implantation of cortical electrodes induces a foreign body response (FBR) in the surrounding brain tissue, ultimately compromising device functionality and long-term stability [6,7].

Multiple cell types contribute to the development of the FBR, including microglia, astrocytes, oligodendrocyte progenitor cells, and vascular-associated cells [8,9,10]. Among these, astrocytes play a central role in the formation of the peri-implant glial scar, which can mechanically and electrically isolate the electrode from surrounding neurons [11]. During scar formation, astrocytes undergo astrogliosis characterized by changes in gene expression, cytoskeletal organization, and cellular morphology [12,13]. Upregulation of glial fibrillary acidic protein (GFAP) is widely recognized as a hallmark of reactive astrocytes in both foreign body response and diverse CNS pathologies [14,15]. Furthermore, a limited number of studies have reported that quantitative parameters of GFAP-stained astrocyte morphology can serve as markers of the cell functional state and brain tissue pathology outcomes [16,17]. In contrast to purely qualitative assessments, quantitative analysis of astrocyte morphology and GFAP signal provides a set of measurable parameters that reflect astrocyte functional states and tissue pathology. Therefore, quantification of the astrocytic GFAP expression and cell morphology can be viewed as a promising readout of the severity of the FBR caused by neuroimplantation and, more generally, for neuroimplant biocompatibility studies.

Reliable morphometric analysis requires automated and unbiased image processing pipelines that minimize operator-dependent variability. Machine learning-based image segmentation has become a widely adopted strategy for biomedical microscopy analysis over the past two decades [18,19]. Deep learning approaches demonstrate high performance but typically require large annotated datasets and substantial computational expertise, limiting their accessibility for routine laboratory use [20,21]. With respect to astrocytes in brain sections, deep learning-based segmentation tools have not yet become widely adopted, although several highly promising pioneering studies were reported [22,23].

Shallow learning approaches provide an attractive alternative for applications with limited ground truth data. Several user-friendly implementations enable efficient cell segmentation with minimal training data and rapid classifier generation [24,25]. In supervised and shallow learning-based image segmentation, the quality and structure of training annotations are known to critically influence classifier performance. Recent studies in medical image analysis emphasize that segmentation accuracy and generalization remain strongly dependent on annotation quality, inter-observer variability, and dataset design, particularly in settings with heterogeneous signal intensity and ambiguous boundaries [26,27,28]. However, in the context of GFAP-based astrocyte morphometry, the role of annotation strategy has not been systematically addressed.

In the present study, we took advantage of the LabKit plugin for Fiji, which is based on a random forest classifier and integrates seamlessly into reproducible image analysis pipelines [25,29]. It has been successfully applied to segmentation of diverse microscopy datasets including cultured cells, bacterial images, tissue sections, large light-sheet volumes, and 3D ultrastructural datasets [25,30,31,32]. We demonstrate the peri-implant scar-specific changes in astrocyte morphology, GFAP expression, and subcellular distribution. We further compare several classifiers with various astrocyte segmentation outcomes and propose a procedure for generating robust classifiers applicable to repetitive and independent experiments. We also provide an explicit validation of annotation strategies for shallow learning-based astrocyte segmentation.

## 2. Results

**Figure 1:**
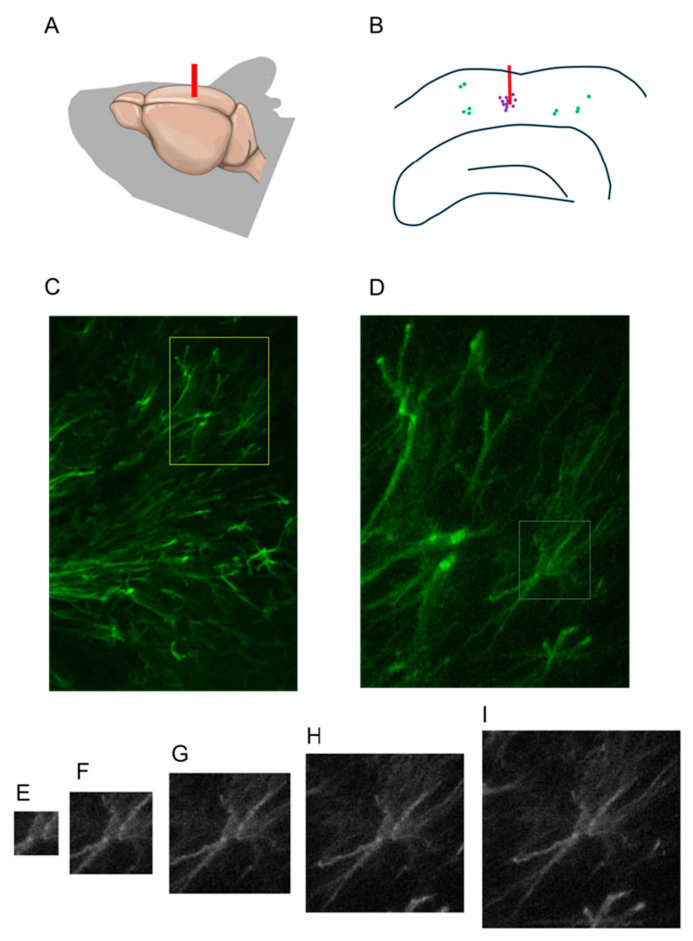
Astrogliosis within foreign body response to neuroimplantation. (**A**) Schematic representation of cortical electrode implantation. (**B**) Scar-resident (violet) and non-resident (green) astrocytes in a mouse brain section. (**C**) GFAP-positive astrocytes within the glial scar at the implantation site. (**D**) An enlarged region of interest corresponding to the yellow rectangle in C. (**E**) A single astrocyte, shown in a white rectangle in D. (**E**–**I**) Selection of an optimal square size for single cell image analysis.

We first asked whether the recently developed advanced algorithm for shallow learning-based image segmentation, LabKit [25], can be used for the segmentation of GFAP-stained astrocytes in histological sections of mouse brain with implantation-induced scarring. At the 2-month time point after implantation, cortical sections exhibited pronounced GFAP-positive scars at the implantation sites (Figure 1A–D). The GFAP-positive astrocytes were tightly packed within the scar (Figure 1C,D). In addition to that, some sparse GFAP-positive astrocytes could be observed in the cortex areas away from the implantation sites.

We then used the semi-automatic procedure for single-cell perineuronal net analysis [33] and adjusted it for picking single astrocytes in squares of the desired size (Figure 1E–I).

### 2.1. Creation of Classifiers

**Figure 2:**
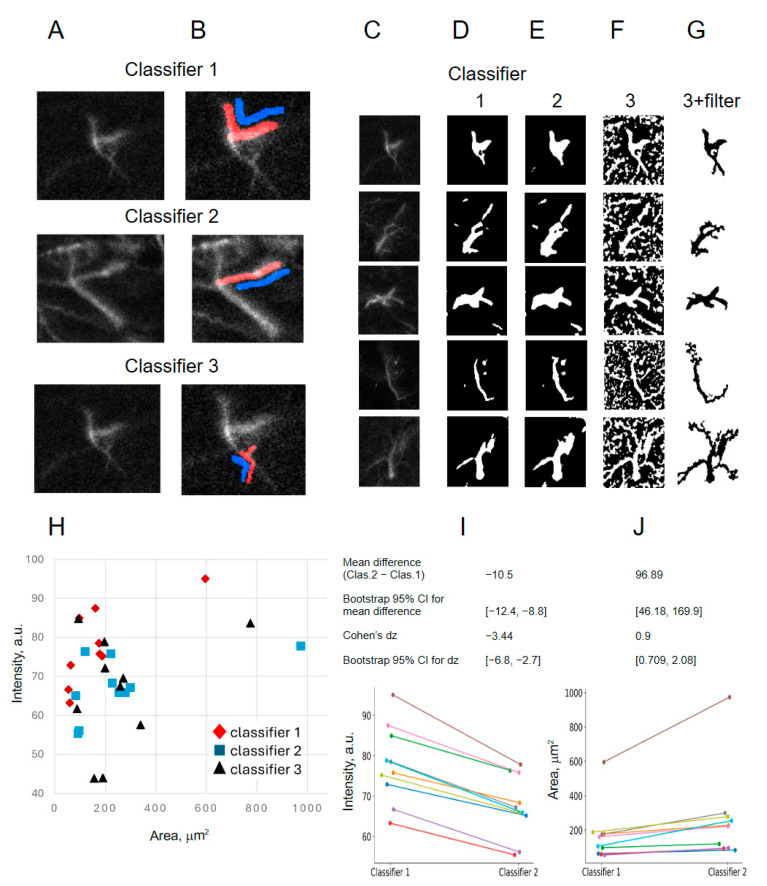
Training and comparison of Labkit classifiers in Fiji software. (**A**) Original cell images used for shallow learning. (**B**) Foreground (red) and background (blue) selection for shallow learning. (**C**) Original images for testing the classifiers. (**D**–**F**) Masks by classifiers 1–3, respectively. (**G**) Object size threshold-filtered masks for classifier 3. (**H**) Single cell intensity-area scatter plot for 10 cells, classifiers 1–3. (**I**) Classifier 1 produces systematically higher mean GFAP fluorescence intensity results as compared to classifier 2. Data shown for the same 10 cells as in H. (**J**) Classifier 1 produces systematically lower GFAP-positive cell area results as compared to classifier 2.

We created a few LabKit classifiers on astrocytes within and away from the peri-implantation cites (Figure 2A,B) to check how robustly the algorithm would perform on cells of various sizes, shapes, and GFAP intensity levels (Figure 2C–G). One crucial advantage of the LabKit shallow learning method is that it takes a skilled human researcher less than a minute to create a single classifier that can be then tested on many astrocytes. The classifiers 1 and 2 segmented mostly large GFAP-positive objects corresponding to the astrocytic cells with few or no small peripheral fragments (Figure 2C–E). By contrast, classifier 3 segmented large numbers of small objects in the analyzed area (Figure 2C,F). We performed extensive trials to find an optimal object threshold size to filter away many small noisy fragments without compromising the main tree of astrocyte processes (Figure 2G).

### 2.2. Fiji Pipelines with Embedded Machine Learning

The Labkit plugin was designed with implicit capacity for integration in Fiji pipelines and automated processing of big data [25]. To build on those features, we assembled two pipelines for semi-automatic and automated processing of the brain section confocal images. Pipeline 1 works on raw microscopy images. It starts by manual selection of a cell (region of interest, ROI) center with the PointPicker tool followed by manual selection of the ROI size (Figure 1E–I). Multiple cells (ROIs) can be selected within a single pipeline run. The astrocyte-containing ROI is then segmented with a pre-defined Labkit classifier, the resulting mask is filtered for the object size, and the minimum operation is applied between the raw ROI and the filtered mask. The cell mean intensity, area, perimeter, area-to-perimeter ratio, and circularity (isoperimetric) are written to the output files for further analysis. The set of the quantified parameters can be easily expanded as needed. Pipeline 2 works on a pre-selected set of ROIs, performs Labkit-based segmentation with subsequent intensity and morphometry analysis in the same way as in pipeline 1. On average, this approach improves the speed of the image analysis by 5.2 times and profoundly reduces the number of manual operations for the whole procedure.

### 2.3. Testing the Classifiers

The results of astrocyte segmentation performed by those classifiers exhibited consistently higher cell areas and lower mean GFAP intensities for some classifiers as compared to others (Figure 2H–J). This result suggests that LabKit classifiers can be created specifically either for the brightest GFAP cytoskeleton area (the cell “core”) or for larger and dimmer areas reflecting more closely the astrocyte area in 2D images and the astrocyte volume in 3D.

**Figure 3:**
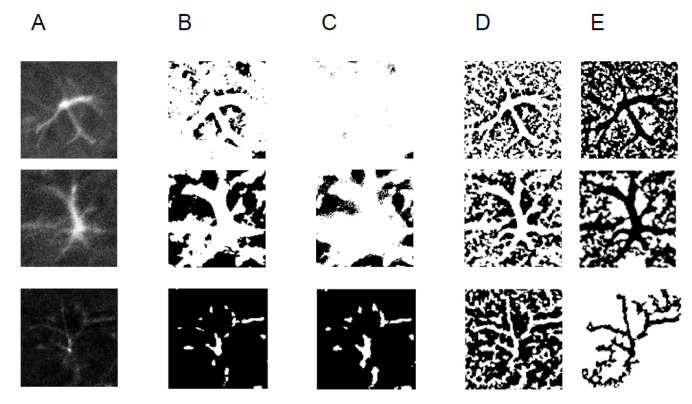
Testing Labkit classifiers on repeated and independent experiments. (**A**) Original images for 3 astrocytes. (**B**–**D**) Applying classifiers 1–3 (shown in Figure 2) to astrocytes from another mouse reveals systematic failure of cell border segmentation. (**E**) Object size threshold-filtered masks for classifier 3.

Efficient and standardized image processing implies that data from repetitive independent experiments are subjected to exactly the same image analysis procedure. Therefore, it would be crucial that we develop LabKit classifiers equally applicable to astrocyte images from independent experiments. Testing classifiers 1–3 on confocal images from different mice (three independent experiments) revealed that some cells were not segmented correctly (Figure 3). More specifically, classifiers 1 and 2 produced very blurred segmentation masks (Figure 3A–C), while classifier 3 segmented almost the entire analyzed image as an uninterrupted syncytium of processes (Figure 3A,D,E) that does not reflect the true astrocyte morphology previously described by various independent techniques in glial scars [12,30].

### 2.4. Selection of the Classifiers

**Figure 4:**
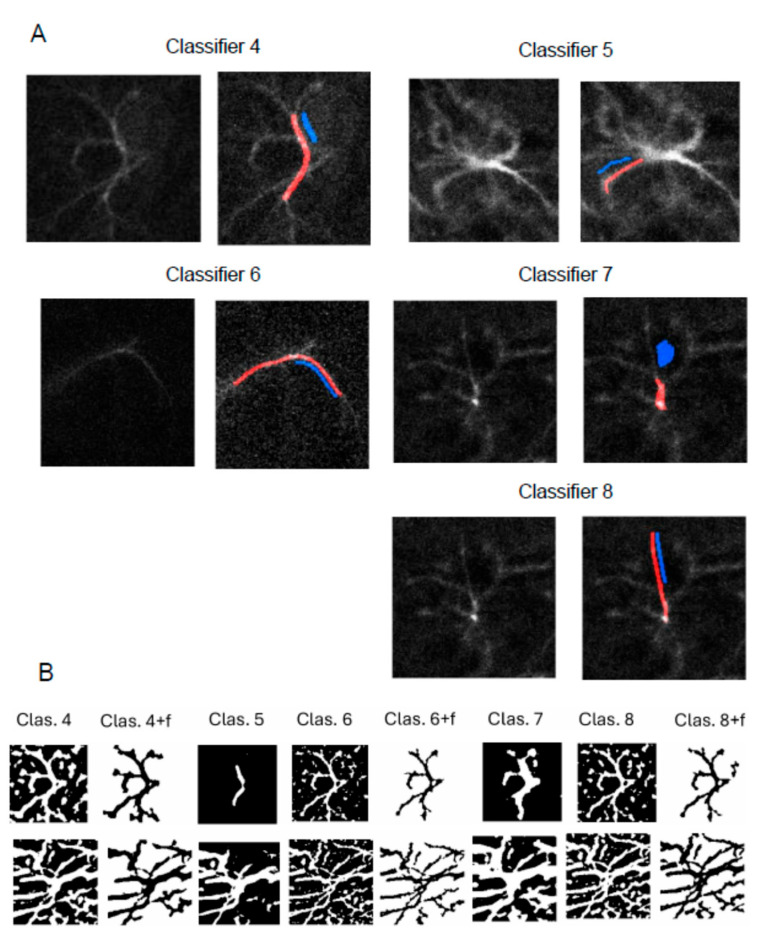
Comparison and selection of Labkit classifiers. (**A**) Five additional classifiers (numbers 4–8) were trained and tested with different patterns of the foreground (red) and background (blue) selection. (**B**) Comparison of segmentation results showed that classifiers 4, 6, and 8 systematically performed more successfully as compared to classifiers 5 and 7 on images from repeated and independent experiments.

To overcome the limited applicability of the LabKit shallow learning approach to repetitive and independent experiments, we developed an extended panel of the LabKit classifiers producing different astrocyte segmentation results (Figure 4). Altogether, we tested 100 cells from four mice (six brain sections, four independent experiments) processed with eight different classifiers. Those extensive trials allowed us to select three classifiers (numbers 4, 6, and 8), robustly and consistently performing astrocyte segmentation in microscopy data from independent experiments.

### 2.5. Training Rules for Successful Classifiers

**Figure 5:**
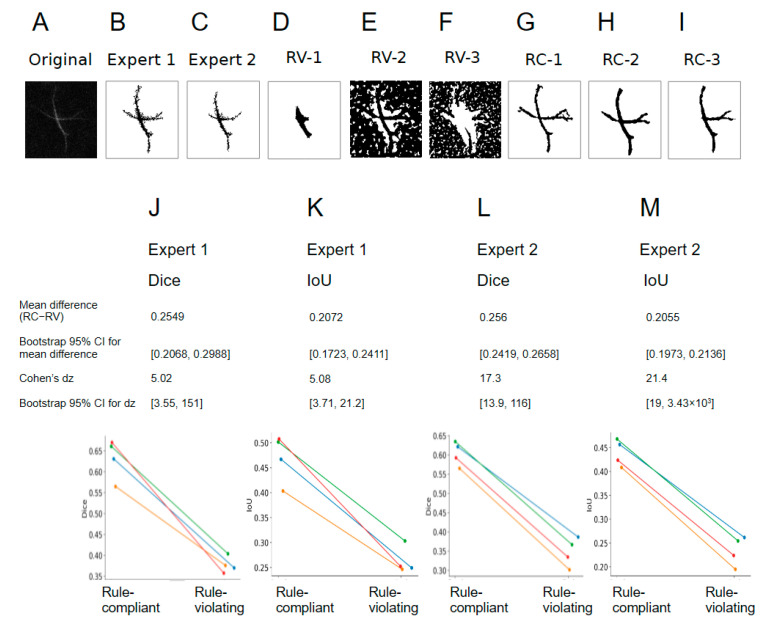
Shallow learning training rules affect segmentation performance. A testing dataset of 25 astrocytes from 4 mice (an example in (**A**)) was segmented by two skilled experts (intensity threshold masks, examples in (**B**,**C**)), three rule-violating (RV) classifiers (**D**–**F**), and three rule-compliant (RC) classifiers (**G**–**I**). Two different match indices—the Dice coefficient and Intersection over Union (IoU)—were calculated, and the values were averaged across the three rule-compliant and three rule-violating classifiers (**J**–**M**). Data are presented as per-animal averages for 4 mice (N = 4). The dataset included at least 5 cells from each mouse, for a total of 17 scar-resident and 8 non-scar-resident cells.

Careful comparison of the foreground and background sampling (the red and blue lines in Figure 2 and Figure 4) made it possible to suggest some “rules of thumb” for the best classifier creation: (1) classifiers 4, 6, and 8 were made on cells from outside the scars; (2) the foreground line was traced through the cell body and the longest process of an astrocyte; (3) the foreground line contained both bright and dim pixels; and (4) the foreground and the background lines were traced along and next to each other. To further validate this set of rules, we created six additional classifiers: three classifiers following the rules and three classifiers trained against the rules (Figure S1). Match indices (Dice coefficient and Intersection over Union (IoU)), calculated between expert annotation masks and classifier-derived masks, were consistently higher for the three rule-compliant classifiers as compared to the three rule-violating classifiers on a dataset of 25 astrocytes from four mice (Figure 5).

### 2.6. GFAP Expression and Astrocyte Morphology as Quantitative Markers of the Foreign Body Response

After selecting the best classifiers, we asked whether the LabKit-assisted segmentation results can reveal specific quantitative difference in the GFAP expression between the scars and distant astrocytes outside the scars. The magnitude of standardized effects was interpreted using conventional benchmarks (small ≈ 0.2, medium ≈ 0.5, and large ≈ 0.8) originally proposed by Cohen [34] and discussed for paired designs by Lakens [35]. Across all three classifiers, cell area and perimeter measurements consistently indicated astrocyte hypertrophy in scar regions.

**Figure 6:**
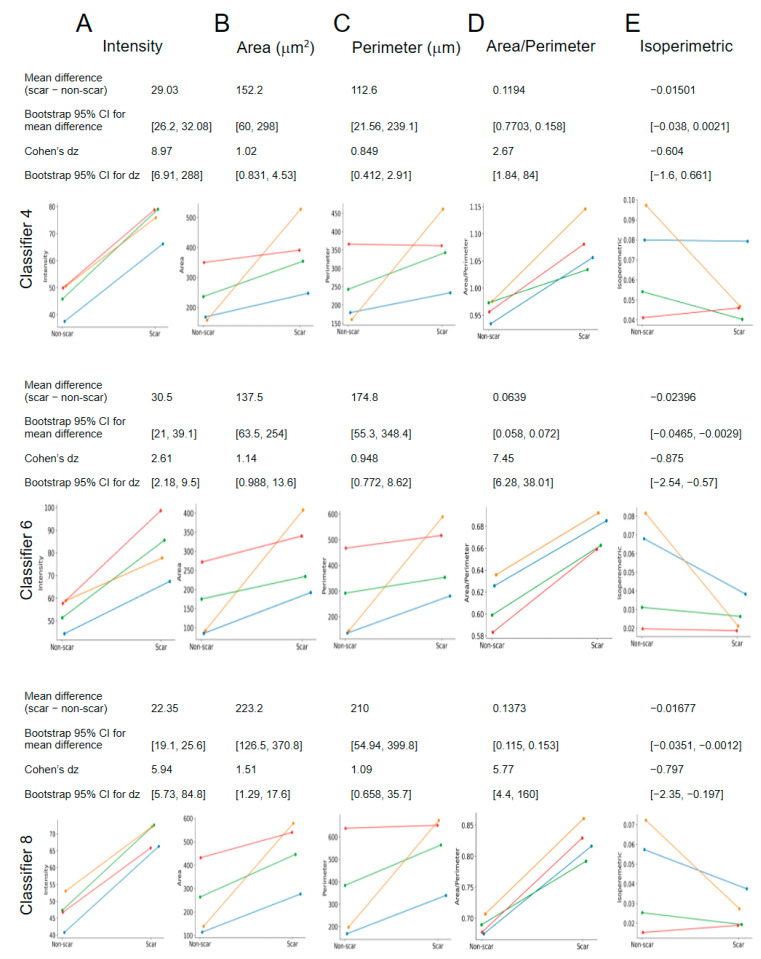
Comparison of GFAP intensity and morphometry parameters for scar-resident versus non-resident astrocytes. (**A**) GFAP intensity. (**B**) Cell area. (**C**) Cell perimeter. (**D**) Area-to-perimeter ratio. (**E**) Cell circularity (isoperimetric). Data are shown as per-animal averages for 4 mice (N = 4, with a total of 100 cells) for three classifiers (Nos. 4, 6, and 8). The same set of 100 cells was analyzed using all three classifiers.

GFAP intensity was increased in scar tissue in all classifiers, with mean paired differences ranging from 22.35 to 30.5 and bootstrap 95% confidence intervals consistently excluding zero. Standardized effect sizes were large to very large (Cohen’s dz ranging from 2.61 to 8.97), indicating a robust elevation of GFAP signal across animals (Figure 6A).

Cell area was likewise elevated in scar regions, with mean paired differences ranging from 137.5 to 223.2 µm^2^ and bootstrap confidence intervals consistently above zero. Effect sizes were large across classifiers (dz = 1.02–1.51), supporting pronounced astrocyte hypertrophy (Figure 6B).

Cell perimeter showed a similar pattern, with mean paired differences between 112.6 and 210 µm and large standardized effect sizes (dz = 0.849–1.09), consistent with expansion of astrocytic cell contours (Figure 6C).

The area-to-perimeter ratio was increased in scar regions in all classifiers (mean paired differences 0.0639–0.1373), with large to very large effect sizes (dz = 2.67–7.45), indicating systematic alteration of astrocyte geometry (Figure 6D).

The isoperimetric index, in contrast, showed a modest decrease in scar regions (mean paired differences −0.015 to −0.024). While effect sizes were moderate in magnitude (dz = −0.60 to −0.88), confidence intervals indicated greater variability across classifiers compared to other parameters (Figure 6E).

Taken together, the three independent classifiers demonstrated strong gross consistency in detecting astrocyte hypertrophy and geometric remodeling in scar regions, despite moderate quantitative variation in specific shape descriptors.

### 2.7. GFAP Expression in the Astrocytic Processes

Astrocyte processes are a crucial cellular compartment performing highly important physiological functions within the blood–brain barrier [36], regulation of synaptic transduction and plasticity [37], and the “palisade” formation with the glial scars [13]. Being a cytoskeleton component, GFAP is expected to bear a structural function and to hold a role in shaping the astrocyte process tree. Therefore, GFAP expression in astrocytic processes may be considered as a promising distinct marker of the astrocyte functional state [38]. We first assessed what part of the whole process tree is grasped by the Labkit masks. By comparing total process length in the Labkit masks versus raw images from a random data subset, we estimate that Labkit reveals 83% of all processes on average (Figure 6A–C).

We then used the LabKit-assisted segmentation results to measure GFAP expression specifically in the astrocytic processes. At the level of astrocytic processes, classifier 4 revealed consistent alterations in scar regions.

**Figure 7:**
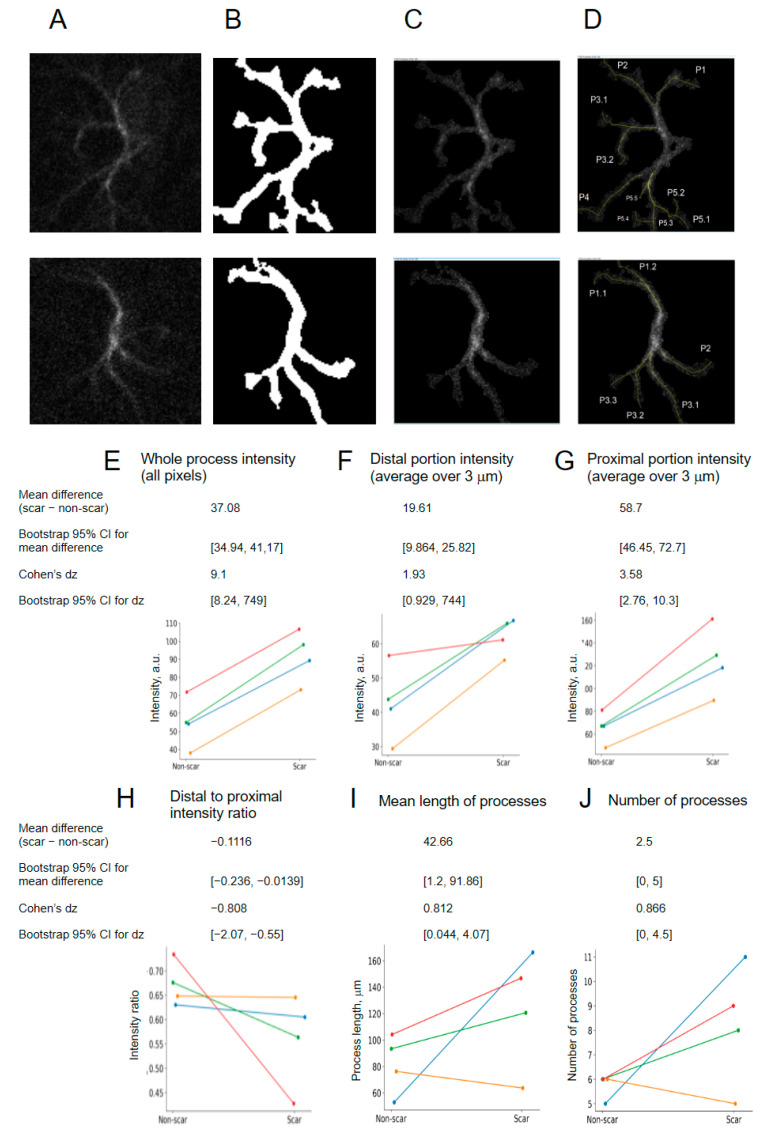
GFAP upregulation and redistribution in the processes of the scar-resident astrocytes based on Labkit-assisted segmentation. (**A**). Original images for 2 astrocytes. (**B**) Masks by the classifier 4. (**C**) Result of the Fiji Image Calculator “minimum intensity” operation applied to images (**A**,**B**). (**D**) Astrocytes with traced processes. (**E**–**H**) GFAP intensity redistribution upon scarring. (**I**) Process length measurements. (**J**) Number of processes measurements. Data are shown for classifier 4 as per-animal averages for 4 mice (N = 4, with a total of 100 cells).

Whole-process GFAP intensity (all pixels) was markedly increased in scar tissue (mean paired difference = 37.08; bootstrap 95% CI: 34.94–41.17). The standardized effect size was very large (Cohen’s dz = 9.1; bootstrap 95% CI: 8.24–749), indicating a highly consistent elevation of signal along astrocytic processes (Figure 7E).

When spatial compartments were analyzed separately, both distal and proximal portions exhibited increased intensity. Distal process intensity increased with a mean paired difference of 19.61 (bootstrap 95% CI: 9.864–25.82), corresponding to a large effect size (dz = 1.93; bootstrap 95% CI: 0.929–744) (Figure 7F). Proximal process intensity showed an even larger increase (mean paired difference = 58.7; bootstrap 95% CI: 46.45–72.7), with a very large, standardized effect size (dz = 3.58; bootstrap 95% CI: 2.76–10.3) (Figure 7G).

Consistent with this asymmetric increase, the distal-to-proximal intensity ratio was reduced in scar regions (mean paired difference = −0.1116; bootstrap 95% CI: −0.236 to −0.0139). The effect size was moderate to large (dz = −0.808; bootstrap 95% CI: −2.07 to −0.55), indicating relative proximal enrichment of GFAP signal (Figure 7H).

Regarding structural parameters, mean process length was increased in scar regions (mean paired difference = 42.66; bootstrap 95% CI: 1.2–91.86), with a large, standardized effect size (dz = 0.812; bootstrap 95% CI: 0.044–4.07) (Figure 7I). The number of processes also tended to increase (mean paired difference = 2.5; bootstrap 95% CI: 0–5), with a large effect size (dz = 0.866; bootstrap 95% CI: 0–4.5), although confidence intervals indicated greater variability (Figure 7J).

Taken together, process-level analysis demonstrated robust increases in GFAP signal intensity and process elaboration in scar regions, accompanied by relative proximal enrichment of cytoskeletal signal.

## 3. Discussion

Foreign body response remains a major factor limiting the long-term performance of intracortical neuroimplants [5,39]. Astrocytes are key contributors to this response through the formation of a dense glial scar that alters tissue mechanics, electrical properties, and neuron–electrode coupling [6,7]. In the present study, we demonstrate that shallow machine learning-assisted segmentation of GFAP immunofluorescence images provides a practical and quantitative approach for assessing implantation-induced astrogliosis at both whole-cell and process levels. Beyond its specific application to neuroimplantation, the workflow described here may also be useful for quantitative analysis of astrocyte morphology across diverse neuropathological contexts.

A major methodological challenge in this context is that GFAP-based astrocyte morphometry is highly sensitive to segmentation strategy, particularly in conventional tissue sections where astrocyte boundaries are not directly visualized and GFAP signal spans bright cores together with dimmer processes. The present study was therefore designed to quantify implantation-induced astrogliosis in a reproducible manner and to assess how different classifier training strategies affect the results of shallow learning-based segmentation for astrocyte morphometry.

Astrogliosis represents a dynamic and graded response. Early stages involve reversible GFAP upregulation and moderate morphological changes, whereas more severe injury can lead to persistent structural remodeling and scar formation [12,14,40]. The increased GFAP expression and morphological differences between scar-resident and distant astrocytes observed in this study are therefore consistent with distinct stages of astrocyte activation.

A key finding of our work is that LabKit-based random forest classifiers can reliably segment GFAP-positive astrocytes in confocal images of tissue samples without the need for extensive training datasets. This accessibility is particularly important because GFAP immunostaining remains one of the most widely used approaches for evaluating neuroinflammation in a broad range of brain pathologies and neuroimplant biocompatibility. In contrast to deep learning approaches, which require large, carefully curated ground truth datasets and specialized programming expertise with the involvement of computational specialists, the shallow learning framework implemented in LabKit offers a favorable balance between performance, accessibility, and reproducibility. Classifier training can be performed rapidly by biomedical researchers without programming expertise, making the approach well suited for iterative exploratory analysis and routine laboratory workflows. An additional practical advantage of the present workflow is that the entire analysis pipeline—including cell selection, ROI definition, segmentation, process-level annotation, and morphometric quantification—was implemented within a single Fiji-based environment. This integration facilitates reproducible analysis and reduces the need for data transfer between different software platforms, which can otherwise complicate and slow down the quantitative analysis of large numbers of cells. Importantly, this practical accessibility enables systematic comparison of multiple classifier configurations and annotation strategies within a consistent analytical framework, which was essential for achieving the aims of the present study.

At the initial stage of this work, we encountered a critical limitation: classifiers trained on one experimental dataset often failed to generalize to astrocytes from independent experiments (Figure 3). This observation highlighted the lack of robustness and transferability of segmentation models without further methodological refinement. The systematic investigation of annotation strategies presented here was therefore directly motivated by this problem. By refining annotation rules and training procedures, we were able to substantially improve classifier performance across independent datasets (Figure 4, Figure 5 and Figure 6), thereby establishing a more robust and reproducible segmentation workflow.

Our systematic comparison of multiple classifiers revealed that segmentation outcomes strongly depend on the foreground and background sampling strategy used during training. Some classifiers preferentially captured only the brightest GFAP-positive structures corresponding to the astrocytic “core,” whereas others included extended and dimmer process networks. This variability indicates that different classifiers capture distinct subsets of morphological features within the same cell image dataset, suggesting that classifier design can be tuned to emphasize specific biological aspects of cell morphology. Importantly, by testing classifiers across independent experiments and animals, we identified a subset of classifiers that performed robustly despite inherent variability between experiments. From this analysis, we derived practical “rules of thumb” for classifier generation, including training on astrocytes outside the scar, sampling both bright and dim GFAP regions, and closely juxtaposing foreground and background annotations.

Importantly, these guidelines were subsequently validated by generating additional rule-compliant and rule-violating classifiers, demonstrating that classifiers trained according to the proposed annotation strategy consistently achieved higher segmentation success rates. This result indicates that in shallow learning–based segmentation workflows, the design of the annotation strategy plays a critical role in determining classifier performance. To our knowledge, systematic validation of annotation strategies for shallow learning-based astrocyte segmentation has rarely been addressed explicitly. Thus, an important practical outcome of the present work is not merely the use of a particular plugin but the establishment of an explicit annotation framework that can be adopted, tested, and refined in future studies.

In the broader field of biomedical image segmentation, annotation variability is increasingly recognized as a key factor influencing model performance and reproducibility [27,28]. At the same time, recent advances highlight that segmentation outcomes remain sensitive to dataset design, annotation protocols, and domain-specific variability [26,41]. However, these issues have been primarily discussed in the context of deep learning and large-scale datasets, whereas in shallow learning-based workflows, and particularly in GFAP-based astrocyte morphometry, annotation strategy has received comparatively little explicit attention. Our results extend this line of reasoning by demonstrating that even within a fixed algorithmic framework, differences in annotation strategy can lead to systematically distinct segmentation outcomes. This suggests that annotation design should be considered an integral component of segmentation workflows, rather than a purely technical preprocessing step.

Using the selected robust classifiers, we detected increased GFAP expression in scar-resident astrocytes (Figure 6). This observation is consistent with multiple qualitative and semi-quantitative reports describing GFAP upregulation as a hallmark of reactive astrogliosis in foreign body response and other CNS injuries [42,43,44]. Importantly, our approach moves beyond binary or region-based assessments of GFAP intensity and instead enables single-cell-resolved quantitative analysis. Such cell-level resolution is essential for dissecting the heterogeneity of astrocyte responses within and around the glial scar.

Morphometric analysis revealed concordant enlargement of astrocytes in scar regions, including increased cell area and perimeter together with elongation of GFAP-positive processes. These findings are consistent with classical descriptions of reactive astrocyte hypertrophy involving cell body enlargement and remodeling of the process arbor [34,37]. Notably, the morphometric parameters of cell area and cell perimeter used in this study should be interpreted as measures of the GFAP-positive cytoskeletal domain rather than the full astrocyte morphology. Notably, the circularity shape descriptor exhibited greater variability across classifiers than size-related metrics. This observation indicates that parameters reflecting boundary geometry are more sensitive to segmentation differences and should therefore be interpreted cautiously. At the same time, such variability may help distinguish robust hypertrophic changes from features that depend more strongly on segmentation assumptions.

A particularly novel aspect of this study is the analysis of GFAP expression within astrocytic processes. Astrocyte processes form the primary interface with synapses, vasculature, and other glial elements and contribute critically to synaptic regulation, blood–brain barrier function, and scar organization [37,38]. Notably, the relative distribution of GFAP signal within astrocytic processes appeared shifted toward proximal compartments in scar regions, as indicated by a reduced distal-to-proximal intensity ratio (Figure 7). To our knowledge, this quantitative pattern has not been explicitly assessed before; however, it is consistent with previously reported qualitative descriptions of cytoskeletal remodeling and proximal enrichment of intermediate filaments in reactive astrocytes [34,45]. The observed decrease in the distal-to-proximal ratio may reflect increased local mechanical rigidity, redistribution of intermediate filaments toward sites of mechanical load, or formation of a structural “support core” around the implant accompanied by loss of thin distal processes [46,47]. These findings may be particularly relevant for understanding how astrocytes mechanically and biochemically contribute to electrode encapsulation.

The approach described here complements existing high-resolution 3D astrocyte reconstruction techniques, which provide exquisite morphological detail but are labor intensive, low throughput, and often rely on transgenic or viral labeling strategies. A recent study by Faust and coauthors implemented Labkit for that kind of high-resolution astrocyte imaging data analysis [48]. By contrast, our method is applicable to conventional GFAP immunostaining and low-resolution imaging, allowing for rapid analysis of large numbers of samples. This trade-off favors scalability and translational relevance, especially in studies aiming to compare implant materials, surface modifications, or pharmacological interventions across experimental cohorts.

Several limitations should be considered. GFAP labels only a subset of astrocytic cytoskeletal structures and does not capture the full extent of fine perisynaptic processes. Therefore, the metrics reported here may be relevant for quantification of the implant-induced scarring but not for accurately addressing whole-cell morphology. The presented pipeline is designed to perform several important steps in an automated manner. Nevertheless, the method remains susceptible to observer-dependent bias, as it relies on manual selection of the cell center and ROI size. Ideally, the operator should be unaware of whether the tissue originates from scar or non-scar regions. In practice, however, complete blinding is difficult to achieve because these regions are readily distinguishable based on astrocyte density. The observer-dependent bias could be partially reduced by selecting the cell square size in a blinded manner (Figure 1). To test this option, we performed additional blinded control analyses that further validated our experimental procedure (Figure S2). High cell density in scar regions may introduce an additional source of bias due to overlapping astrocytic processes. In such cases, processes from neighboring astrocytes may be included within the selected ROI as small peripheral objects, which are subsequently removed using size-based filtering. However, complete exclusion of overlap between process arbors of adjacent cells cannot be guaranteed. Additionally, the number of analyzed cells was constrained by the density of GFAP-positive astrocytes outside the scar and by the availability of well-defined scar regions. Nevertheless, the methodological framework described here is readily scalable and could be applied to hundreds or thousands of cells in future studies. Finally, while we focused on GFAP as a canonical marker of astrogliosis, the same workflow could be extended to other astrocytic markers or combined with multiplexed imaging approaches.

In summary, shallow machine learning-assisted image analysis provides a reproducible and accessible framework for quantitative assessment of astrocyte responses to neuroimplantation and a broad range of neuroinflammatory conditions. By enabling consistent single-cell and subcellular measurements across experiments, this approach supports high-throughput evaluation of foreign body response and has the potential to facilitate the development of strategies to improve neuroimplant biocompatibility. Our results demonstrate that annotation strategy represents an important determinant of segmentation outcomes in shallow learning-based workflows. The combination of shallow learning segmentation and explicit annotation rules may therefore help standardize astrocyte morphometry across studies of neuroinflammation. This may be particularly valuable for comparative analyses of implant materials, surface modifications, and anti-inflammatory interventions, as well as for broader applications in CNS injury and disease, where robust and transferable quantification of astrocyte responses is required.

## 4. Materials and Methods

### 4.1. Animals

All experimental procedures involving animals complied with Directive 2010/63/EU of the European Parliament and Council, as well as Order No. 267 of the Ministry of Health of the Russian Federation (19 June 2003). The study protocol was approved by the Commission on Biological Safety and Bioethics of ITEB RAS (Pushchino; approval No. 40/2023, 15 February 2023).

### 4.2. Electrode Implantation

Stereotaxic surgeries were carried out under general anesthesia using isoflurane (1–4%). Nichrome electrodes (50 μm diameter) were implanted into the primary visual cortex (V1) at the following stereotaxic coordinates: AP = 2.8 mm, ML = 2.5 mm, and DV = 0.5 mm.

### 4.3. Immunohistochemistry

Eight weeks after surgery, animals were deeply anesthetized and perfused transcardially with 4% paraformaldehyde prepared in 0.1 M phosphate buffer (pH 7.4). Brains were extracted and post-fixed in the same solution. Coronal sections (35 μm thickness) were obtained using a Leica VT1200 S vibratome (Leica Microsystems, Wetzlar, Germany).

Free-floating sections were subjected to double labeling for GFAP (anti-chicken, ab4674, Abcam, Cambridge, UK) and perineuronal nets using Wisteria floribunda agglutinin (Vector Laboratories, Newark, CA, USA). All staining procedures were performed in 24-well plates.

Sections were rinsed three times in PBS containing 0.5% Triton X-100 (PBS-T, pH 7.4), followed by incubation in blocking solution (1% bovine serum albumin in PBS-T) for 1 h. Endogenous biotin was blocked using a commercial Streptavidin/Biotin Blocking Kit (Vector Laboratories, USA), according to the manufacturer’s instructions.

Primary reagents (biotinylated WFA, 1:500; anti-chicken GFAP, 1:500) were diluted in 10 mM HEPES-buffered PBS (pH 7.4), and sections were incubated overnight at 4 °C. After three washes (5 min each) in PBS-T, sections were incubated with Alexa Fluor 633–conjugated streptavidin (Invitrogen; 1:100) (Thermo Fisher Scientific, Waltham, MA, USA) for 30 min, followed by washing. Subsequently, sections were incubated for 2 h with goat anti-chicken secondary antibody conjugated to Alexa Fluor 488 (A11039; 1:500), Invitrogen, Thermo Fisher Scientific, Waltham, MA, USA. After final washes, sections were mounted on glass slides, air-dried, and coverslipped.

### 4.4. Microscopy

Images were acquired using a Leica TCS SP5 confocal microscope (Leica, Germany). Imaging was performed with an oil immersion objective (HC X PL APO CS 20×/0.70 IMM UV; Leica Microsystems, Wetzlar, Germany) at a pixel size of 300 nm.

### 4.5. Image Analysis

Image processing and analysis were conducted using FIJI (software version ImageJ 1.54p) [29]. Astrocyte segmentation was performed using the random forest-based LabKit plugin, enabling implementation of a reproducible workflow integrated with subsequent morphometric analysis [25]. This setup also allowed for systematic comparison of classifiers trained using different annotation strategies across datasets.

Segmentation performance was evaluated using overlap-based metrics, including the Dice coefficient and Intersection over Union (IoU). Dice was defined as 2|A ∩ B|/(|A| + |B|) and IoU as |A ∩ B|/|A ∪ B|, where A represents the expert annotation and B the classifier output.

Astrocytic processes were manually traced using the Line tool. Fluorescence intensity profiles were extracted along each traced process. Mean intensity values were calculated for (i) the entire process, (ii) the distal segment (last 10 pixels, i.e., 3 μm), and (iii) the proximal segment (first 10 pixels, i.e., 3 μm).

### 4.6. Statistical Analysis

Data were analyzed using estimation statistics, reporting mean paired differences with bootstrap-derived confidence intervals, rather than relying exclusively on null-hypothesis significance testing [49,50].

Standardized paired effect sizes (Cohen’s dz) were calculated to quantify within-animal differences [35,36].

For the analyses presented in Figure 6 and Figure 7, statistical comparisons were based on per-animal averages (4 mice; N = 4 independent experiments). Cell-level measurements were averaged for at least 10 astrocytes per condition (scar-resident and non-scar-resident) per animal, yielding a total of 100 cells across 4 mice.

## Data Availability

Data are available upon request.

## Supplementary Materials

The following supporting information can be downloaded at: https://www.mdpi.com/article/10.3390/ijms27083524/s1.
