# Supplementary figures and images for: Foreign Body Response to Neuroimplantation: Machine Learning-Assisted Quantitative Analysis of Astrogliosis
Source: Int J Mol Sci. 2026 Apr 15;27(8):3524. doi: 10.3390/ijms27083524 (PMC13115741; doi:10.3390/ijms27083524)

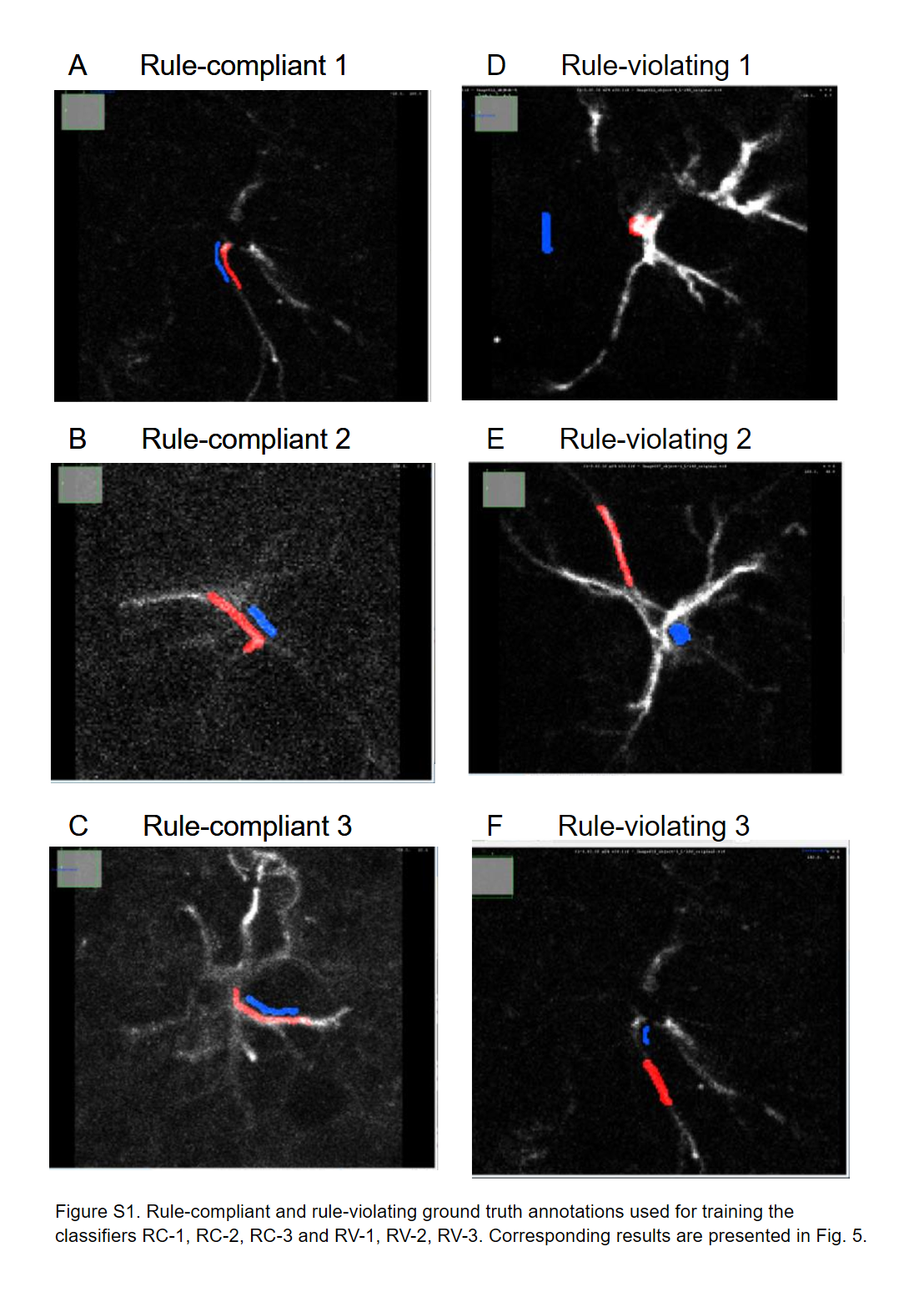

Supplement: Supplementary file 1 [file ijms-27-03524-s001.zip › Fig S1 astrocyte 3.4.bmp]

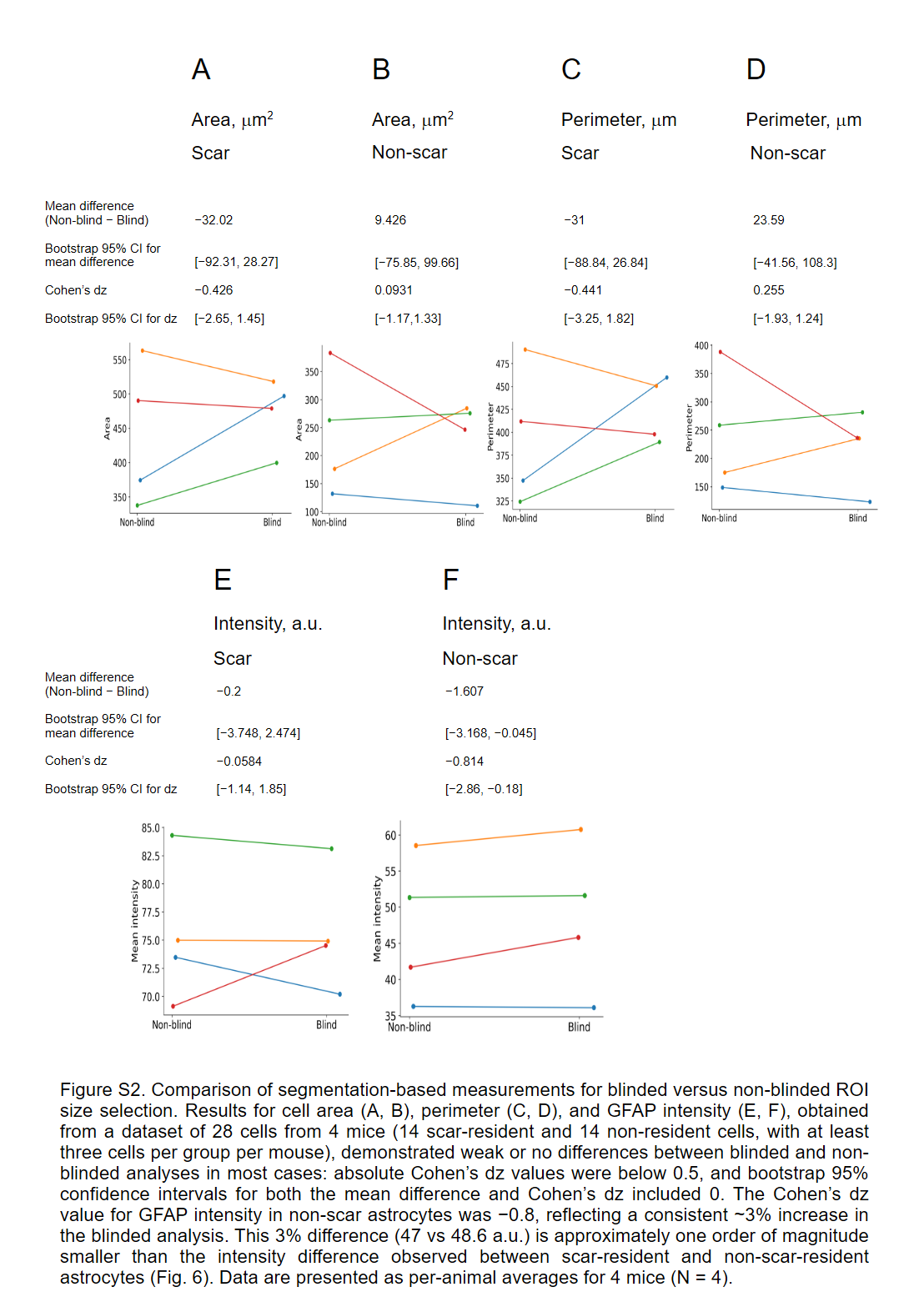

Supplement: Supplementary file 1 [file ijms-27-03524-s001.zip › Fig S2 astrocyte implant 14.4.bmp]
